# Antioxidant homeostasis is disturbed in fetuses with leptin-resistant genotypes: A cross-sectional study

**Published:** 2018-08

**Authors:** Pedro Gonzalez-Añover, Laura Torres-Rovira, Consolacion García-Contreras, Marta Vazquez-Gomez, Jose Luis Pesantez, Maria Victoria Sanz-Fernandez, Susana Astiz, Antonio Gonzalez-Bulnes

**Affiliations:** 1 *Faculty of Veterinary Sciences, UCM, Madrid, Spain.*; 2 *Comparative Physiology Group, SGIT-INIA, Madrid, Spain.*; 3 *School of Veterinary Medicine and Zootechnics, Faculty of Agricultural Sciences, University of Cuenca, Cuenca, Ecuador.*

**Keywords:** Animal models, Fetus, Leptin, Oxidative stress, Pregnancy

## Abstract

**Background:**

Leptin resistance is associated with lower reproductive efficiency, with deficiencies in embryo viability and growth leading to low prolificacy and high incidence of intrauterine growth restriction.

**Objective:**

We aimed to investigate the underlying mechanisms of the leptin-resistance, evaluating the antioxidant homeostasis of leptin-resistant and lean swine fetuses.

**Materials and Methods:**

The study included 70 plasma samples from fetuses at day 62 of gestation (mid-pregnancy), from breeds with (Iberian breed; n=35) and without leptin resistance (25% Large White x 25% Landrace x 50% Pietrain; n=35). The antioxidant status of the plasma samples was determined by photoinduced chemiluminescence whilst systemic oxidative stress was assessed determining plasma hydrogen peroxide concentration by enzimoimmunoassay.

**Results:**

Plasma total antioxidant capacity was significantly lower in leptin-resistant fetuses (p=0.003), whilst systemic oxidative stress was increased (p=0.02).

**Conclusion:**

Our results indicate key differences in the antioxidant status in pregnancies affected by leptin resistance.

## Introduction

Obesity is linked to reproductive disorders (1, 2). During pregnancy, changes in the maternal physiological status induce alterations in the intrauterine environment where the conceptus evolves, modifying its homeostasis. As a result, the fetal development may be hampered by either excessive- or restricted-growth (intrauterine growth restriction or IUGR), which leads to large-or small-for-gestational-age newborns, respectively. In both cases, offspring life-long health and fitness are affected by developmental programming (3). Robust and reliable translational models are essential in order to investigate the underlying mechanisms. The most amenable large-animal model for obesity is the pig (4) and there is a swine breed, the Iberian pig, which is a specific model for studies on obesity associated with leptin resistance (5, 6). 

The Iberian pigs are homozygous for a leptin receptor gene polymorphism similar to the leptin resistance syndrome described in humans (7). The Iberian breed is also characterized by a lower reproductive efficiency compared to the lean swine breeds, showing deficiencies both in embryo viability and growth, which lead to a low prolificacy and to a high incidence of IUGR when compared to lean breed sows (8). However, fetuses with leptin-resistant genotype also have a high resilience to adverse conditions, through improved developmental and metabolic adaptations (9). Nevertheless, after birth developmental programming can affect the offspring by inducing higher corpulence and adiposity and the development of metabolic disorders, being similar to humans (10). There is increasing evidence which demonstrates that obesity compromises maternal and feto-placental antioxidant status and redox balance (11, 12). Moreover, the production of reactive oxygen species (ROS) and subsequent oxidative stress are hypothesized to be one of the key early mediators of the offspring programming (11). However, there is a scarcity of knowledge on the possible effects of the leptin resistance syndrome on pregnancy outputs in dams with adequate diet and absence of obesity. 

Hence, the present study aimed to evaluate whether differences in the antioxidant homeostasis may be a mechanism determining the viability and growth of Iberian fetuses by comparing them to lean crossbred fetuses (i.e.: comparing genotypes with and without leptin resistance) exposed to the same nutritional and environmental conditions (i.e.: avoiding maternal obesity).

## Materials and methods

This cross-sectional study involved blood samples from 70 fetuses of the CPG-INIA biobank. Fetuses were obtained from 7 Iberian (group IB; n=35 fetuses) and 6 lean crossbred sows (25% Large White × 25% Landrace × 50% Pietrain; group L, n=35 fetuses) at day 62 of gestation (mid-pregnancy), Such time-point was chosen because fetal metabolism in mid-pregnancy is mainly driven by genetic traits whilst it is more affected by nutrient requirements and availability at later stages (13). All the females were nulliparous, twelve months old, and were inseminated for purebred litters. During the experimental period, the sows were individually fed with a standard grain-based diet individually adjusted to body-weight to fulfill the basal requirements for the pregnancy status, avoiding obesity. 

At day 62 of pregnancy, the conceptuses were recovered and, immediately, blood samples were drawn from the heart and/or umbilical cord with heparinized syringes, centrifuged at 1500 g for 15 min and frozen into polypropylene vials until assay. The fetuses were sexed, weighed and classified as IUGR when weight was under one SD of the mean litter weight value. Plasma antioxidant status was determined by photoinduced chemiluminescence (Minilum®, ABCD GmbH, Berlin, Germany) with an ARAW©-series kit from ABCD GmbH, Berlin, (Germany). Systemic oxidative stress was assessed using hydrogen peroxide as a marker, and its plasma concentration was measured by enzimoimmunoassay (Abcam, Cambridge, UK).

**Ethical consideration**

The females from both groups were housed together in collective pens at the INIA Animal Unit and managed under Project Licenses approved by the INIA Committee of Ethics in Animal Research (reports CEEA 2010/003 and CEEA 2013/036).

**Statistical analysis**

SPSS software (Statistical Package for the Social Sciences, version 22.0, SPSS Inc, NY, IBM, USA) was used for statistical analysis. Effects of fetal breed, sex and normal/IUGR status on antioxidant capacity and oxidative stress were assessed by analysis of variance (ANOVA) corrected for variance homogeneity by Bonferroni test and *post hoc* Duncan test. The results were expressed as mean±SEM and statistical significance was accepted from p<0.05.

## Results

**Figure 1:**
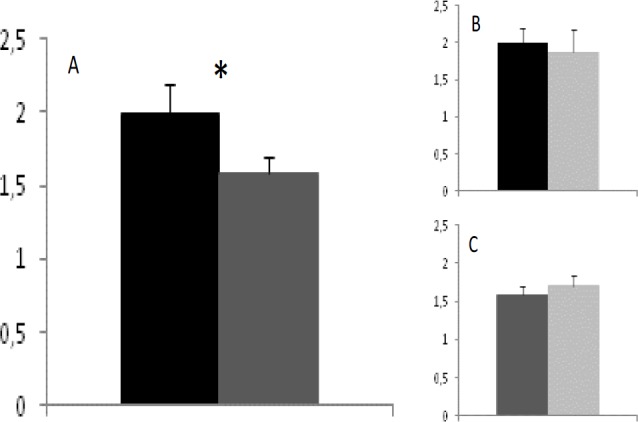
Total antioxidant capacity (µmol/ml ± S.E.M.) of plasma from lean crossbreed and Iberian purebred fetuses (black and dark-grey bars, respectively; panel A), and effects of normal/ IUGR status (black/dark-grey and light grey, respectively; panel B for lean crossbred and panel C for Iberian fetuses). Asterisk indicates significant differences (p=0.003).

**Figure 2:**
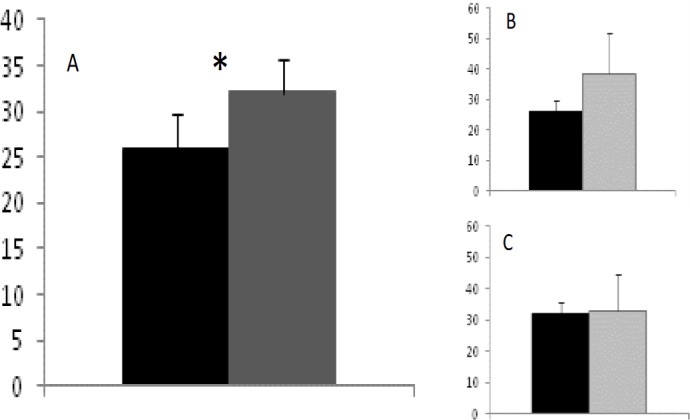
Systemic oxidative stress (hydrogen peroxide concentrations in plasma; µmol/ml ± S.E.M.) of lean crossbreed and Iberian purebred fetuses (black and dark-grey bars, respectively; panel A), and effects of normal/ IUGR status (black/dark-grey and light grey, respectively; panel B for lean crossbred and panel C for Iberian fetuses). Asterisk indicates significant differences (p=0.02).

Plasma antioxidant capacity was significantly lower in IB than in L fetuses (Figure 1; p=0.003), without significant effects of sex within the breed. The assessment of plasma hydrogen peroxide concentrations showed significantly increased production in IB fetuses (Figure 2; p=0.02), with similar values in male and female fetuses within each breed. The comparison between fetuses with adequate development and fetuses affected by IUGR showed no significant differences in the IB breed, but IUGR fetuses from the L group showed a trend (p=0.07) for a lower plasma antioxidant capacity. The determination of plasma hydrogen peroxide showed higher values in IUGR fetuses of both breeds, but the high individual variability of IUGR precluded statistical significance.

## Discussion

The results of the present study, using a swine model of obesity due to leptin resistance (Iberian pig) indicate that the fetuses from leptin-resistant genotypes have a decreased antioxidant capacity and, in consequence, a higher systemic oxidative stress than lean crossbred fetuses. These results indicate, by the first time, a link between dysfunctions in leptin pathways and deficiencies in the redox system.

To the best of our knowledge, our results are the first to evidence that fetal antioxidant homeostasis is compromised in leptin-resistant genotypes, resulting in increased oxidative stress, even in the absence of maternal obesity. In general, leptin-resistant genotypes are affected by low reproductive efficiency, with affected individuals being prone to infertility (1). Currently, from the results of in vitro assisted reproductive protocols, the role of failures in implantation and adequate prenatal development is getting more and more important, since individuals with leptin resistance evidence deficiencies both in embryo viability and growth, which lead to a low fertility and a high incidence of prenatal growth restriction (8). Leptin and its receptor are found at the endometrium (14-16) and are involved in implantation and fetal development (17, 18). Deficiencies in vascular growth and irrigation of placentation sites, finally causing hypoxia, preeclampsia, and IUGR are found in case of abnormalities in this pathways (19).

Moreover, hypoxia would increase oxidative stress and would aggravate the effects of IUGR, as found in fetuses exposed to maternal hypobaric hypoxia (20). The main consequence of deficiencies in antioxidant capacity and increased oxidative stress during pregnancy is increased lipids peroxidation. Increased lipid peroxidation, in turns, decreases lipid availability at the fetoplacental unit; mainly of polyunsaturated fatty acids (PUFA; 21-24). Finally, impaired placental transfer of PUFA causes or exacerbates IUGR (23, 25, 26).

The results of the present study give in vivo evidence supporting the existence of a link between energy metabolism and redox homeostasis (27), which converges at the mitochondria level. In the classical picture, the primary signal of energy status is the hormone leptin, which is produced in the adipocytes and communicates the levels of energy stores to the arcuate nucleus in the hypothalamus. Upon activation, the leptin/melanocortin hypothalamic pathway regulates appetite and satiety, energy homeostasis and adipose tissue metabolism (28). However, there is increasing evidence addressing the existence of other leptin-regulated arcuate-independent pathways at different organs and tissues. In these pathways, leptin would act at the mitochondria, through the transcription factors 5’-AMP-activated kinase (AMPK) and the nuclear factor-kB (NF-kB). Thus, the mitochondria would be a key organelle for energy metabolism. The mitochondria would be at the center of the redox homeostasis as well; AMPK also regulates oxidative stress through changes in NF-kB activation. In consequence, when modulating the energy balance, the mitochondria is also regulating ROS production and oxidative stress (27). This hypothesis seems to be confirmed in our study; dysfunctions in leptin pathways would be linked to deficiencies in the redox system. 

In addition, our results confirm previous evidence of a higher resilience of the Iberian fetuses to adverse environmental conditions (9). In the lean crossbred genotype, in spite of the high individual variability, the IUGR status was associated with a trend for decreased antioxidant capacity status and a higher ROS production, similar to earlier studies indicating exacerbated oxidative stress in IUGR pregnancies (29). The values were almost equal when comparing normal and IUGR Iberian fetuses, which in addition to the developmental and metabolic adaptations (9) would favor vitality and survival rates of the offspring in this breed. However, in both genotypes, parameters for antioxidant capacity and oxidative status were similar in both male and female fetuses, evidencing no significant sex-related effects; which supports previous findings in the Iberian breed (30).

## Conclusion

The present results indicate key differences in the antioxidant status in pregnancies affected by leptin resistance, independently of obesity, and set the basis not only for more specific studies on the extent and role of the oxidative stress within the pathophysiology of the leptin resistance syndrome but also for the development of specific therapies.
